# Characteristics and HIV epidemiologic profiles of men who have sex with men and transgender women in key population-led test and treat cohorts in Thailand

**DOI:** 10.1371/journal.pone.0203294

**Published:** 2018-08-30

**Authors:** Pich Seekaew, Supabhorn Pengnonyang, Jureeporn Jantarapakde, Thanthip Sungsing, Piyanee Rodbumrung, Deondara Trachunthong, Chun-liang Cheng, Thitiyanun Nakpor, Ratchadej Reankhomfu, Danai Lingjongrat, Surang Janyam, Sutinee Charoenying, Stephen Mills, Michael Cassell, Praphan Phanuphak, Ravipa Vannakit, Nittaya Phanuphak

**Affiliations:** 1 Thai Red Cross AIDS Research Centre, Bangkok, Thailand; 2 Sisters Foundation, Chonburi, Thailand; 3 Caremat Organization, Chiang Mai, Thailand; 4 Rainbow Sky Association of Thailand, Bangkok, Thailand; 5 The Service Workers in Group Foundation, Bangkok, Thailand; 6 FHI 360 and USAID LINKAGES Project, Bangkok, Thailand; 7 Office of Public Health, U.S. Agency for International Development Regional Development Mission Asia, Bangkok, Thailand; National and Kapodistrian University of Athens, GREECE

## Abstract

Men who have sex with men (MSM) and Transgender Women (TGW) in Thailand contribute to more than half of all new HIV infections annually. This cross-sectional study describes epidemiologic profiles of these key populations (KP) in Key Population-led Test and Treat study. Baseline data were collected using self-administered questionnaires and HIV/STI testing from MSM and TGW aged ≥18 years enrolled in a cohort study in six community sites in Thailand between October 2015 and February 2016. Factors associated with HIV prevalence were determined by logistic regression. TGW in the cohorts had lower education and income levels than MSM. TGW also engaged in sex work more, though similar proportions between MSM and TGW reported to have multiple sexual partners and STI diagnosis at baseline. HIV prevalence was 15.0% for MSM and 8.8% for TGW in the cohorts. HIV prevalence among TGW was more associated with sociodemographic characteristics, whereas factors related to behavioral risks were determined to be associated with HIV prevalence among MSM. TGW and MSM in the cohorts also had high prevalence of STI. Key Population-driven HIV services are able to capture harder-to-reach key populations who are at heightened risk for HIV.

## Introduction

In 2015, UNAIDS estimated that there were 5.1 million people in Asia living with HIV and 300,000 new HIV infections, making it the second highest HIV prevalence and incidence in the world [1]. Thailand, along with nine other countries, accounted for 96% of all new infections in the region, with the Key Populations (KP) consisting of men who have sex with men (MSM), sex workers, people who inject drugs (PWID), and transgender women (TGW) [2]. Though Thailand sharply reduced HIV incidence among heterosexual population in the early period of the epidemic [3, 4], the country has been experiencing a plateau in the decline of HIV incidence in recent years [5], with viral transmission continues to disproportionately affect MSM and TGW populations [6]. They contribute to more than half of all new HIV infections in Thailand [6], and HIV prevalence and incidence among Thai MSM and TGW in major cities are worrisome, compared to HIV prevalence among general population of 1.1% [5]. In 2014, the estimated number of MSM in Thailand was 532,646 [7], and the median HIV prevalence among MSM in Thailand was 9.2% [5]. Further, it was determined that MSM in Bangkok had HIV prevalence of 28.6%, making it the highest in Asia-Pacific region [2]. Similarly, the incidence among MSM in Bangkok was reported to be 5 to 6 per 100 persons-year based on the observational and synthetic cohort studies conducted during the same period [5]. The approximation of TGW population in Thailand in 2014 was 314,808 [7], with the median HIV prevalence of 11.7% [5]. HIV incidence among TGW could not be reported, as the data was not available.

These numbers represent the severity of HIV condition in Thailand, urging for novel actions to alleviate the epidemic. WHO has called for differentiated approaches to delivering HIV care to KP, including MSM and TGW, emphasizing the need to create enabling environments to increase accessibility and acceptability of HIV services [8].

In Thailand, KP members have played vital roles in addressing the epidemic, and their partnership with multilateral aid agencies has changed the landscape of HIV care. There are 4 key Community-Based Organizations (CBOs) who work with MSM (Service Workers in Group Foundation (SWING)), TGW (Sisters), and MSM with TG-specific services integrated (Rainbow Sky Association of Thailand (RSAT), and Caremat). The Thai Red Cross AIDS Research Centre (TRCARC) has worked with these CBOs, with funding support from The U.S. President’s Emergency Plan for AIDS Relief (PEPFAR) through the U.S. Agency for International Development (USAID)’s Linkages Across the Continuum of HIV Services for Key Populations Affected by HIV (LINKAGES) project, to build KP-led Health Service (KPLHS) capacities. KPLHS are a defined set of HIV-related health services that focus on specific KPs, and are delivered by CBOs, run by KPs, in partnership with other health sector entities.

This study aims to describe demographic and behavioral characteristics of MSM and TGW, as well as their HIV epidemiologic profiles, enrolled in Key Population-led Test and Treat cohorts and were offered health services through KPLHS model in Thailand.

## Methods

### Study design and participants

In 2015, we initiated a prospective observational cohort study for the Key Population-led Test and Treat model with six community-based clinic sites in Bangkok, Chiang Mai, Chonburi, and Songkhla. We have partnered with six gender-specific community health centers (RSAT Bangkok, SWING Bangkok, SWING Pattaya, Sisters, Caremat, and RSAT Songkhla) to implement “Test and Treat” strategy, aiming to increase uptake of HIV/STI services among Thai MSM and TGW, and initiate ART early regardless of CD4 count among those infected with HIV. Potential participants were recruited in the study geographical areas through enhanced KPLHS outreach activities, including: incentivized case findings (a peer-to-peer communication and support system), promoting through social media channels frequented by MSM/TGW, working with the MSM and TGW “community influencers,” setting up information table in high MSM/TGW prevalent areas, and peer referral. Clients who accessed HIV counseling and testing services at each community-based site were recruited by clinic counselors and study staff. Furthermore, informative pamphlets regarding the study were distributed by community outreach workers in local areas. We recruited eligible MSM and TGW who were aged 18 years and older, were Thai nationals, had sex with men, had condomless anal intercourse with a man in the last six months or had at least three male sex partners in the last six months, were not known to be HIV-positive, and signed informed consent. Eligible MSM and TGW were enrolled during May 2015 to October 2016, and followed for 24 months. In this study, 2,646 participants were eligible, and were invited to join the study. All participants provided informed consented before proceeding with study procedures.

The study was approved by: Chulalongkorn University Institutional Review Boards (Community-Based Test and Treat IRB: 181/57), The Ethics Committee for Research in Human Subjects Department of Diseases Control (IRB:9/57-678), Queen Savang Vadhana Memorial Hospital (IRB:21/2557), The Research Ethics Committee of Hatyai Hospital (IRB: 53/2560), Chonburi Provincial Public Health Office (IRB: cb0032.003/658), and Chiang Mai Provincial Public Health Office (cm0032.003.1/6609); NCT Number: NCT02383602.

### Study sites

#### Rainbow Sky Association of Thailand (RSAT)

Rainbow Sky Association of Thailand was established in 1998, and was one of the first CBOs to serve gay male population affected by HIV/AIDS in Thailand. There are 7 RSAT sites around the country; however, only RSAT Bangkok and RSAT Songkhla were included in this study. Through a mix of face-to-face outreach, social media, and hotspot-based outreach, RSAT is able to reach approximately 10,000 MSM and TGW into the service each year. RSAT provides Voluntary Counseling and Testing (VCT) for HIV on site, and offers mobile HIV testing at hotspot areas. Moreover, RSAT offers Pre-Exposure Prophylaxis (PrEP) and Post-Exposure Prophylaxis (PEP) to its clients, in addition to condoms and lubricants. RSAT community health workers also link those who are confirmed HIV seropositive to point-of-care CD4, as well as provide psychosocial support and ART adherence counseling.

#### Service Workers in Group Foundation (SWING)

Service Workers in Group Foundation was established in 2004, focusing its services on sex workers in Thailand. SWING is located in Bangkok and Pattaya, cities with large sex-driven entertainment venues. SWING provides HIV testing and syphilis screening to its clients. PrEP and PEP are also available at the drop-in-centers. And for those who are HIV-infected, SWING offers point-of-care CD4 count, emotional and psychosocial support, as well as ART adherence counseling.

#### Sisters

Sisters was established in 2004 with an aim to provide comprehensive services specifically to TGW. Sisters is located in Pattaya, Chonburi, and its services include: counseling on gender affirming healthcare, HIV testing, syphilis screening, and point-of-care CD4 count. Further, Sisters provides mobile HIV testing at hotspot areas frequented by TGW.

#### Caremat

Caremat was established in 2003, and is located in Chiang Mai, a Northern part of Thailand. The facility provides HIV testing and syphilis screening, as well as PrEP and PEP to its clients.

### Procedures

#### Physiological assessment

Pre-test and post-test counselings, including risk reduction counseling, were provided to participants according to the standard practice at each site. All participants received HIV test at baseline. Those who tested HIV-negative were asked to come for HIV re-testing every six months or sooner if they felt exposed to risk. Participants newly diagnosed with HIV were offered ART immediately regardless of CD4 count. Sexually transmitted infections (STIs) were screened regardless of symptoms, and nucleic acid amplification test (NAAT) was used to test for gonorrhea and chlamydia on pharyngeal swab, urine, rectal swab, and neovaginal swab (only for TGW who had undergone sexual reassignment surgery). Treponema Pallidum Haemagglutination (TPHA) with Venereal Disease Research Laboratory (VDRL) or Rapid Plasma Reagin (RPR) confirmation were used to diagnose syphilis. Treatment for STIs was provided to participants. Support for partner notification was also provided.

#### Questionnaires

Self-administered questionnaires were given to participants at baseline visit to capture information on demographic profiles, behavioral risks, and HIV knowledge prior to HIV testing. In addition to the baseline visit, questionnaires pertaining behavioral risk were asked at every follow-up visit.

### Statistical analysis

Statistical analysis was conducted with Stata version 14.1 (Statcorp, College Station, TX, USA).

The demographic characteristics of the participants, together with their baseline behavior risk information, STI and HIV clinical characters, were reported overall and by gender-specific groups (MSM and TGW) as: frequency and proportion for categorical variables; mean, standard deviation (SD), median and interquartile range (IQR) for continuous variables. Comparison of continuous variables between groups was made by using two-sample t test or Mann-Whitney U test. Chi-square or Fisher’s exact was used for comparison of proportion of characteristics between groups.

HIV prevalence was assessed at baseline and 95% Confidence Interval (95%CI) around the prevalence rate which was calculated according to a binomial distribution. The difference in HIV prevalence between groups was tested by Chi-square.

Binary logistic regression was used to explore correlations between factors and HIV prevalence. Assumptions about linearity of continuous covariates such as age, age at first sex, number of sexual partners were checked by breaking the variable into quartiles and examining the odds ratio (OR) and 95% CI for each quartile. When these assumptions were not met, categorical groupings were used and adjacent quartiles were collapsed together, if appropriate. Baseline covariates with p<0.10 were included and adjusted for in multivariable models by enter method.

## Results

### Demographics of participants

A total of 2,646 participants enrolled in the Key Population-led Test and Treat cohorts. MSM accounted for 70.3% (n = 1,860) and TGW constituted 29.7% (n = 786). There was a significant difference between the age at enrollment between MSM and TGW (mean (SD) = 26.4 (7.3) vs. 25.8 (6.6), p = 0.046). TGW samples were more likely to be single (78.8% vs. 71%, p<0.001), identified as Buddhist (95.7% vs. 92.2%, p = 0.001), have education level less than bachelor degree (83.6% vs. 67%, p<0.001), and engage in sex work (32.3% vs. 19.8%, p<0.001) when compared to MSM counterparts. Moreover, TGW participants had a lower proportion in earning an income of 10,000 Baht per month (35.6% vs. 42.6%, p<0.001) (Table 1).

**Table 1:** Baseline demographic characteristics of MSM and TGW Participants.

Characteristics	Overall (N = 2,646) N (%)	MSM^d^ (n = 1,860) N (%)	TGW^e^ (n = 786) N (%)	P-value
**Age (years)**				
Mean(SD)	26.2(7.1)	26.4(7.3)	25.8(6.6)	0.046^a^
Age 18–25	1475(55.7)	1029(55.3)	446(56.7)	0.50^c^
Age>25	1171(44.3)	831(44.7)	340(43.3)	
**Site**				<0.001^c^
RSAT BKK^h^	639(24.2)	536(28.8)	103(13.1)	
SWING BKK^i^	307(11.6)	289(15.5)	18(2.3)	
SWING PTY^j^	217(8.2)	193(10.4)	24(3.0)	
Sisters	370(14)	61(3.3)	309(39.3)	
Caremat	850(32.1)	565(30.4)	285(36.3)	
RSAT SK^k^	263(9.9)	216(11.6)	47(6.0)	
**Marital status**				<0.001^c^
Single	1940(73.3)	1321(71)	619(78.8)	
Living together with male partner	589(22.3)	434(23.3)	155(19.7)	
Ever been married to a woman	95(3.6)	92(5.0)	3(0.4)	
Missing	22(0.8)	13(0.7)	9(1.1)	
**Religion**				0.001^c^
Buddhism	2466(93.2)	1714(92.2)	752(95.7)	
Others	160(6.0)	131(7.0)	29(3.7)	
Missing	20(0.8)	15(0.8)	5(0.6)	
**Education**				<0.001^c^
Less than bachelor degree	1903(71.9)	1246(67.0)	657(83.6)	
Bachelor degree or above	720(27.2)	594(31.9)	126(16.0)	
Missing	23(0.9)	20(1.1)	3(0.4)	
**Main occupation**				<0.001^c^
Unemployed/student	902(34.1)	663(35.6)	239(30.4)	
Employed	1105(41.8)	814(43.8)	291(37.0)	
Sex workers	622(23.5)	368(19.8)	254(32.3)	
Missing	17(0.6)	15(0.8)	2(0.3)	
**Income(THB**^l^**)**				
Median(IQR)	10000(9000- 15000)	10000(9000- 18000)	10000(9000- 15000)	<0.001^b^
>10,000 Baht/month	1072(40.5)	792(42.6)	280(35.6)	<0.001^c^
Can’t identify/Missing	365(13.8)	266(14.3)	99(12.6)	
**Have ever had HIV testing before enrollment**				0.12^c^
No	1278(48.3)	919(49.4)	359(45.7)	
Yes	1277(48.3)	882(47.4)	395(50.3)	
Missing	91(3.4)	59(3.2)	32(4.0)	
**Have ever used PrEP**^f^				0.42^c^
No	1955(73.9)	1375(73.9)	580(73.8)	
Yes	58(2.2)	45(2.4)	13(1.7)	
Never known PrEP^f^	617(23.3)	428(23.0)	189(24.0)	
Missing	16(0.6)	12(0.7)	4(0.5)	
**Have ever used PEP**^g^				0.13^c^
No	1956(73.9)	1364(73.3)	592(75.3)	
Yes	89(3.4)	71(3.8)	18(2.3)	
Never known PEP^g^	595(22.5)	420(22.6)	175(22.3)	
Missing	6(0.2)	5(0.3)	1(0.1)	

^a^p-value for comparison of mean of characteristic between groups (Two-sample t test)

^b^p-value for comparison of median of characteristics between groups (Mann-Whitney two-statistic)

^c^p-value for comparison of proportion of characteristics between groups (Chi-square test)

^d^MSM: Men who have Sex with Men

^e^TGW: Transgender Women

^f^PrEP: Pre-Exposure Prophylaxis

^g^PEP: Post-Exposure Prophylaxis

^h^RSAT BKK: Rainbow Sky Association of Thailand, Bangkok

^i^SWING BKK: Service Workers in Group Foundation, Bangkok

^j^SWING PTY: Service Workers in Group Foundation, Pattaya

^k^RSAT SK: Rainbow Sky Association of Thailand, Songkhla

^l^THB: Thai Baht

### Sexual and behavioral xharacteristics of participants

TGW in this cohort had their first sexual intercourse at a younger age than their MSM counterparts (median (IQR) = 16 (15–18) vs. 17 (15–19), p<0.001). 49.9% of TGW had their first sex when they were younger than 17 years old (MSM = 38.2%, p<0.001) and 20.1% were younger than 15 years old (MSM = 12.9%, p<0.001). Additionally, TGW were less likely to undergo male circumcision (7.8% vs. 11.5%, p<0.001). When looking at the number of male partners within the past 6 months, TGW reported to have median (IQR) of 2 (2–10) and MSM reported to have 3 (1–5) (p<0.001) (Table 2).

**Table 2:** Baseline behavioral characteristics of MSM and TGW participants.

Characteristics	Overall (N = 2,646) N (%)	MSM^d^ (n = 1,860) N (%)	TGW^e^ (n = 786) N (%)	P-value
**Age at first sexual intercourse(years)**				
Median(IQR)	17(15–19)	17(15–19)	16(15–18)	<0.001^a^
<17	1102(41.7)	710(38.2)	392(49.9)	<0.001^b^
<15	397(15.0)	239(12.9)	158(20.1)	<0.001^b^
Missing	120(4.5)	77(4.1)	43(5.5)	
**Number of sexual partners in the past 6 months**				0.19^b^
No sexual partner/single partner	544(20.6)	392(21.1)	152(19.3)	
Multiple partner	1425(53.9)	1010(54.3)	415(52.8)	
Had sexual intercourse, but refused to answer	667(25.2)	451(24.2)	216(27.5)	
Missing	10(0.3)	7(0.4)	3(0.4)	
**Had sexual intercourse with male in the past 6 months**	2360(89.2)	1611(86.6)	749(95.3)	
Number of male partners, Median(IQR)	3(1–5)	3(1–5)	3(2–10)	<0.001^b^
**HIV perceived risk in the past 6 months**				0.28^b^
Perceived to not have any risk of getting HIV	293(11.1)	197(10.6)	96(12.2)	
Perceived to have risk of getting HIV	2322(87.8)	1640(88.2)	682(86.8)	
Missing	31(1.1)	23(1.2)	8(1.0)	
**Condom used in the past 6 months**^c^				0.86^b^
Sex with condom	565(21.4)	399(21.5)	166(21.1)	
Condomless sex	2042(77.2)	1434(77.1)	608(77.4)	
Missing/Refuse to answer	39(1.4)	27(1.4)	12(1.5)	
**Male circumcision**				<0.001^b^
Yes	275(10.4)	214(11.5)	61(7.8)	
No	1954(73.8)	1390(74.7)	564(71.7)	
Refuse to answer	261(9.9)	151(8.1)	110(14.0)	
Missing	156(5.9)	105(5.7)	51(6.5)	
**Drug used in the past 6 months**				0.79^b^
No	1535(58.0)	1082(58.2)	453(57.6)	
Yes	974(36.8)	685(36.8)	289(36.8)	
Refuse to answer	69(2.6)	46(2.5)	23(2.9)	
Missing	68(2.6)	47(2.5)	21(2.7)	
**Amphetamine-type stimulants used**	168(6.4)	121(6.5)	47(6)	0.62^b^
**Had any symptoms or being diagnosed with any sexually transmitted diseases in the past 6 months**				<0.001^b^
No	1666(63.0)	1159(62.3)	507(64.5)	
Yes	136(5.1)	119(6.4)	17(2.2)	
Not sure	733(27.7)	512(27.5)	221(28.1)	
Refuse to answer	67(2.5)	40(2.2)	27(3.4)	
Missing	44(1.7)	30(1.6)	14(1.8)	
**Sexual transmission infection**				
None	1772/2646(67.0)	1234/1860(66.3)	538/786(68.5)	
Any STI^f^	874/2646(33.0)	626/1860(33.7)	248/786(31.6)	0.29^b^
Syphilis	213/2646(8.1)	185/1860(10.0)	28/786(3.6)	<0.001^b^
Neisseria gonorrhea	379/2627(14.4)	267/1844(14.5)	112/783(14.3)	0.91^b^
Chlamydia trachomatis	558/2627(21.2)	379/1844(20.6)	179/783(22.9)	0.19^b^
**Had group sex in the past 6 months**				0.18^b^
No	2197(83.0)	1532(82.3)	665(84.6)	
Yes	295(11.2)	217(11.7)	78(9.9)	
Missing	154(5.8)	111(6.0)	43(5.5)	

^a^p-value for comparison of median of characteristics between groups (Mann-Whitney two-statistic)

^b^p-value for comparison of proportion of characteristics between groups (Chi-square test)

^c^Condom used: consist of condoms used with HIV infection partners, regular sexual partner, casual sexual partner, commercial sex worker, person who gives you money/goods, sexual partner who is an illicit intravenous drug user in the past 6 months. For participants who did not have sexual intercourse in the past 6 months, we will define these participants as the safe sex subgroup.

^d^MSM = Men who have Sex with Men

^e^TGW = Transgender Women

^f^STI = Sexually Transmitted Infection

### HIV prevalence and associated factors

Of all 1,860 MSM and 786 TGW enrolled in Key Population-led Test and Treat cohort, 329 MSM and 69 TGW tested positive for HIV at baseline. HIV prevalence at baseline was determined to be 15.0% (95%CI: 13.7–16.5); 17.7% (95%CI: 16.0–19.5) among MSM and 8.8% (95%CI:6.9–11.0) among TGW (p<0.001). Factors associated with HIV prevalence among MSM were: residing in Bangkok (OR:2.10; 95%CI:1.48–2.97) and Pattaya (OR:2.17; 95%CI:1.38–3.39), having been married to a woman (OR:0.37; 95%CI:0.15–0.89), never having HIV testing before enrollment (OR:2.61; 95%CI:1.95–3.51), having any STI at baseline (OR:2.91; 95%CI:2.20–3.85), perceiving to have moderate (OR:1.89; 95%CI:1.03–3.47) or high (OR:2.82; 95%CI: 1.49–5.34) risk of acquiring HIV, and being unsure about having any symptoms or being diagnosed with any STI in the past 6 months (OR:1.78; 95%CI: 1.32–2.39) (Table 3). On the other hand, variables associated with HIV prevalence among TGW participants were: being enrolled in the study at age between 18 to 25 (OR:0.51; 95%CI: 0.29–0.89), having less than bachelor degree (OR:4.40; 95%CI:1.31–14.74), never having HIV testing before enrollment (OR:2.16; 95%CI: 1.23–3.77), having any STI at baseline (OR: 3.35; 95%CI: 1.93–5.82), and using amphetamine-type stimulants (ATS) (OR:2.45; 95%CI: 1.05–5.70) (Table 4).

**Table 3:** Logistic regression of factors associated with HIV prevalence for MSM (N = 1860).

Factors	Bivariate			Multivariable		
	OR	95%CI	P-value	OR	95%CI	P-value
**Age (years)**			0.39			
Age>25	1	Ref.				
Age18-25	1.11	0.87–1.41				
**Site**			<0.001			
Chiang Mai	1	Ref.		1	Ref.	
Songkhla	0.79	0.47–1.32		0.95	0.54–1.67	0.86
Pattaya	2.46	1.68–3.60		2.17	1.38–3.39	0.001
Bangkok	1.98	1.46–2.68		2.10	1.48–2.97	<0.001
**Marital status**			0.01			
Single	1	Ref.		1	Ref.	
Living together with male partner	1.11	0.84–1.46		1.20	0.87–1.66	0.26
Ever been married to a woman	0.38	0.17–0.83		0.37	0.15–0.89	0.03
**Education**			0.19			
Bachelor degree and above	1	Ref.				
Less than Bachelor degree	1.19	0.92–1.55				
**Main occupation**			0.45			
Employed	1	Ref.				
Unemployed	0.92	0.70–1.20				
Sex workers (in entertainment venues/restaurants)	1.13	0.83–1.55				
**Income < 10,000 THB**^a^	1.11	0.86–1.44	0.42			
**Never had HIV testing before enrollment**	2.67	2.06–3.47	<0.001	2.61	1.95–3.51	<0.001
**Any STI**^b^	3.77	2.95–4.82	<0.001	2.91	2.20–3.85	<0.001
**Syphilis**	5.26	3.82–7.23	<0.001			
**Neisseria gonorrhea**	3.13	2.35–4.18	<0.001			
**Chlamydia trachomatis**	2.82	2.17–3.66	<0.001			
**Age at first sexual intercourse > 18 years**	0.98	0.76–1.26	0.88			
**Number of sexual partners in the past 6 months**			0.18			
No sexual partner/ Single partner	1	Ref.				
Multiple partners	0.84	0.62–1.14				
Didn’t specify	1.09	0.77–1.54				
**HIV perceived risk in the past 6 months**			<0.001			
No risk	1	Ref.		1	Ref.	
Mild	1.40	0.84–2.33		1.55	0.85–2.83	0.16
Moderate	2.17	1.31–3.59		1.89	1.03–3.47	0.04
High	3.11	1.84–5.24		2.82	1.49–5.34	0.002
**Condom used in the past 6 months**			0.002			0.37
Sex with condom	1	Ref.		1	Ref.	
Condomless sex	1.65	1.19–2.28		1.19	0.81–1.74	
**Male circumcision**			0.22			
No	1	Ref.				
Yes	0.77	0.51–1.17				
**Drug used in the past 6 months**	0.92	0.73–1.15	0.45			
**Amphetamine-type stimulants used**	1.26	0.80–2.00	0.33			
**Had any symptoms or being diagnosed with any STI in the past 6 months**			<0.001			
No	1	Ref.		1	Ref.	
Yes	1.50	0.92–2.43		1.06	0.60–1.85	0.85
Not sure	2.21	1.71–2.87		1.78	1.32–2.39	<0.001
**Had group sex in the past 6 months**			0.08			0.96
No	1	Ref.		1	Ref.	
Yes	1.38	0.97–1.95		1.01	0.67–1.52	

^a^THB = Thai Baht

^b^STI = Sexually Transmitted Infection

**Table 4:** Logistic regression of factors associated with HIV prevalence for TGW (N = 786).

Factors	Bivariate			Multivariable		
	OR	95%CI	P-value	OR	95%CI	P-value
**Age (years)**			0.04			0.02
Age>25	1	Ref.		1	Ref.	
Age18-25	0.59	0.36–0.98		0.51	0.29–0.89	
**Site**			0.19			
Chiang Mai	1	Ref.				
Hat Yai	1.88	0.66–5.36				
Pattaya	1.85	1.01–3.38				
Bangkok	1.74	0.80–3.76				
**Marital status**			0.89			
Living together with male partner	1	Ref.				
Single	1.04	0.55–1.97				
Ever been married to a woman	(omitted)					
**Education**			0.007			0.02
Bachelor degree and above	1	Ref.		1	Ref.	
Less than Bachelor degree	3.35	1.20–9.36		4.40	1.31–14.74	
**Main occupation**			0.11			
Employed	1	Ref.				
Unemployed	0.61	0.31–1.19				
Sex worker (in entertainment venues/restaurants)	1.21	0.69–2.12				
**Income < 10,000 THB**^a^	1.12	0.65–1.93	0.67			
**Have ever had HIV testing before enrollment**	0.50	0.30–0.85	0.009	0.46	0.27–0.81	0.007
**Any STI**^b^	2.96	1.79–4.88	<0.001	3.35	1.93–5.82	<0.001
**Syphilis**	3.01	1.18–0.69	0.04			
**Neisseria gonorrhea**	3.00	1.71–5.23	<0.001			
**Chlamydia trachomatis**	2.92	1.75–4.85	<0.001			
**Age at first sexual intercourse > 18 years**	0.79	0.48–1.31	0.36			
**Number of sexual partners in the past 6 months**			0.03			0.40
No sexual partner/ Single partner	1	Ref.		1	Ref.	
Multiple partners	2.89	1.20–6.92		1.93	0.71–5.23	
Didn’t specify	2.35	0.91–6.02		1.54	0.53–4.47	
**HIV perceived risk in the past 6 months**			0.002			0.25
No risk/Mild	1	Ref.		1	Ref.	
Moderate	2.00	1.11–3.61		1.57	0.82–3.02	
High	3.02	1.61–5.66		1.71	0.84–3.47	
**Condom used in the past 6 months**			0.23			
Sex with condom	1	Ref.				
Condomless sex	1.49	0.76–2.90				
**Male circumcision**			0.82			
No	1	Ref.				
Yes	0.90	0.34–2.34				
**Drug used in the past 6 months**	1.18	0.76–1.83	0.46			
**Amphetamine-type stimulants used**	3.08	1.46–6.50	0.007	2.45	1.05–5.70	0.04
**Had any symptoms or being diagnosed with any STI in the past 6 months**			0.17			
No	1	Ref.				
Yes	2.64	0.73–9.61				
Not sure	1.50	0.88–2.57				
**Had group sex in the past 6 months**			0.37			
No	1	Ref.				
Yes	1.42	0.67–2.99				

^a^THB = Thai Baht

^b^STI = Sexually Transmitted Infection

## Discussion

The findings from this study offer some of the first information illustrating the demographics of MSM and TGW persons using key population-led strategy in Thailand.

We found HIV prevalence (17.7%) among MSM enrolled in the study to be higher than what has been previously reported nationally. In the study, never been tested for HIV, having any STI at baseline, and perceiving to have moderate or high risk of getting HIV were some of the notable factors associated with HIV prevalence. Additionally, HIV prevalence among TGW (8.8%) in these cohorts was also high, and the factors associated with it were also similar to those of MSM (never been tested for HIV and having any STI at baseline). Interestingly, TGW HIV prevalence was also found to be significantly correlated with sociodemographic factors which were absent for MSM counterparts. And these factors were: being enrolled at a young age (18–25), having low education level, and using ATS. This suggests that, in addition to behavioral characteristics, social conditions also attribute to the HIV epidemic among TGW in the cohorts. It is also interesting to note that being married to a woman for MSM and being enrolled in the study at age between 18–25 for TGW were determined to be negative predictors associated with HIV prevalence.

The distinctions between TGW and MSM in the cohorts were also seen when looking at the baseline features of the participants. At the enrollment visit, TGW were relatively younger than MSM, and the majority of them lived outside Bangkok. However, these TGW were still concentrated in populous cities (Chiang Mai and Pattaya). Despite living and working in aforementioned cities, TGW had lower socioeconomic status when compared to MSM, in terms of both education and income levels. Furthermore, higher proportion of TGW engaged in sex work. These differences also echoed in another study conducted in Baltimore, USA, where, when compared to MSM, TGW had a lower education level, were younger, and engaged in injectable drugs more [9]. These sociodemographic differences were not the only qualities distinguishing TGW from MSM in our cohorts, vulnerable behavior exhibitions were also unique to each group. While a significant portion of TGW had their first sexual encounter at a younger age, TGW had a lower number of sexual partners. We also found that condom use during sexual intercourse was lower among TGW than MSM, though this finding was not significant. The study also illustrates alarmingly high rates of STIs among MSM and TGW in the cohorts in which 33.7% and 31.6% of MSM and TGW, respectively, were tested positive for at least one STI, with *Chlamydia trachomatis* (CT) as the most common infection for both MSM (20.6%) and TGW (22.9%). Past literature has reported high prevalence of CT, as well as Neisseria gonorrhoeae (NG), among MSM in Bangkok [10]. Furthermore, our analysis indicates that STI diagnosis was significantly associated with HIV prevalence for both populations. This finding aligns with past study which had determined that rectal CT was significantly associated with HIV acquisition among young MSM in Bangkok [11]. High rates of STIs are quite concerning, especially if they are co-infected with HIV, as it would increase risk of HIV transmission through the increase of viral load in genital discharge [12]. These results strongly suggest that, in addition to HIV testing, STI screening ought to be routinely performed among persons at heightened risk for HIV.

In these cohorts, we found that almost half of MSM (49.4%) and TGW (45.7%) participants were first-time HIV tester. TGW had lower HIV prevalence when compared to that of MSM (8.8% vs. 17.7%), which contradicts previous studies stating TGW were at higher risk of acquiring HIV than MSM (49 times vs 25 times, respectively) when compared to the general populations [13, 14]. HIV prevalence among TGW in our cohorts was lower than those reported in some Southeast Asian settings; for example, Vietnam (18.0%)[15], and Malaysia (12.4%) [16], but slightly higher than TGW cohorts in Cambodia (5.9%) [17]. This variability presents further research questions pertaining unique factors influencing HIV prevalence and incidence among TGW in different settings. Low HIV prevalence in our cohorts may be due to the fact that activities enhancing HIV prevention were tailored to TGW individuals. Targeted HIV prevention program has been showed to increase uptake of HIV testing among TGW in Thailand [18], and our analysis indicates that previous HIV testing has a 54% reduction in the odds of having HIV in this population. Additionally, TGW-specific program was reported to be related to a decrease in number of sexual partners [19]. However, future studies need to assess the unique conditions encompassing Thai TGW.

There are several limitations within this study. Participants enrolled in this study may not be representative of MSM and TGW outside of study settings, as we only selected those who engaged in condomless anal sex, or had three or more partners in the last 6 months, which put them at heightened risk of HIV infection. Thus, the results obtained may not be generalizable and should be interpreted with caution. The outcomes were calculated via cross-sectional analysis, preventing us from analyzing the sociodemographic and behavioral characteristics that may change overtime. Furthermore, we discussed the importance of targeted HIV prevention program for TGW and its associated with HIV prevalence; however, we could not tell if such program would have a significant influence on new HIV infection. Therefore, longitudinal analysis should be done to further investigate how these characteristics change and their effect on HIV incidence. Moreover, information on prior HIV testing was self-reported and we did not validate the answer with official document, and therefore data could be subject to bias.

## Conclusion

Our findings present unique characteristics between Thai MSM and TGW enrolled in Key Population-led Test and Treat cohorts who received sexual health services at CBOs. In addition to behavioral risks, sociodemographic factors were found to be significantly correlated with HIV prevalence among TGW participants; this phenomenon was not observed in MSM. Nonetheless, many participants never received HIV testing prior to being in the study, suggesting that we were able to capture previously harder-to-reach key populations. However, high prevalence of HIV and STI in both populations highlights the urgency for appropriate interventions for these individuals, including routine HIV and STI screening and tailored sexual health program.
